# Transgenerational inheritance of fetal alcohol exposure adverse effects on immune gene interferon-ϒ

**DOI:** 10.1186/s13148-020-00859-9

**Published:** 2020-05-24

**Authors:** Omkaram Gangisetty, Ajay Palagani, Dipak K. Sarkar

**Affiliations:** Rutgers Endocrine Research Program, Department of Animal Sciences, Rutgers University, 67 Poultry Farm Lane, New Brunswick, NJ 08901 USA

**Keywords:** Fetal alcohol, Interferon-ϒ, Epigenetic, Transgenerational transmission

## Abstract

**Background:**

Alcohol exposures in utero have been shown to alter immune system functions in the offspring which persists into adulthood. However, it is not apparent why the in utero alcohol effect on the immune system persists into adulthood of fetal alcohol-exposed offspring. The objective of this study was to determine the long-term effects of fetal alcohol exposure on the production of interferon-ϒ (IFN-ϒ), a cytokine known to regulate both innate and adaptive immunity.

**Methods:**

Isogenic Fisher 344 rats were bred to produce pregnant dams, which were fed with a liquid diet containing 6.7% alcohol between gestation days 7 and 21 and pair-fed with an isocaloric liquid diet or fed ad libitum with rat chow; their male and female offspring were used for the study. F1-F3 generation rats were used when they were 2 to 3 months old. Fetal alcohol exposure effects on the *Ifn-ɣ* gene was determined by measuring the gene promoter methylation and mRNA and protein expression in the spleen. Additionally, transgenerational studies were conducted to evaluate the germline-transmitted effects of fetal alcohol exposure on the *Ifn-ɣ* gene.

**Results:**

Fetal alcohol exposure reduced the expression of *Ifn-ɣ* mRNA and IFN-ϒ protein while it increased the proximal promoter methylation of the *Ifn-ɣ* gene in both male and female offspring during the adult period. Transgenerational studies revealed that the reduced levels of *Ifn-ɣ* expression and increased levels of its promoter methylation persisted only in F2 and F3 generation males derived from the male germ line.

**Conclusion:**

Overall, these findings provide the evidence that fetal alcohol exposures produce an epigenetic mark on the *Ifn-ɣ* gene that passes through multiple generations via the male germ line. These data provide the first evidence that the male germ line transmits fetal alcohol exposure's adverse effects on the immune system.

## Introduction

Maternal alcohol ingestion during pregnancy increases the risk of potentially serious health problems during the early developmental period, even in full-term infants [1–5]. Hospital stays during the first year of life are often longer for infants with fetal alcohol exposure compared with matched control infants, with pneumonia being one of the main reasons for hospitalization [6]. The impact of fetal alcohol exposure on immune functions of an adult human is not well studied. Animal studies show that fetal alcohol has a direct effect on specific aspects of the immune system. Studies of animals exposed in utero to ethanol suggest that ethanol-induced immune dysfunction, particularly impaired innate and adaptive immunity, persists into adulthood [7–9]. Additionally, it has been shown that in utero ethanol exposure increases the response of the hypothalamic–pituitary–adrenal axis, which in turn results in hyperactivity in stress-induced immunosuppression and increased vulnerability to subsequent infectious illness [10, 11]. In fetal alcohol-exposed animals, the reduced release of a stress-regulatory hypothalamic peptide proopiomelanocortin (POMC) is connected to some of the immune abnormalities, particularly reduced natural killer (NK) cell functions and cytokine interferon-γ (IFN-γ) production during the adult period [12, 13].

IFN-γ or type II interferon is a cytokine that participates in innate and adaptive immunity against viral, some bacterial, and protozoal infections. IFN-γ is produced and secreted by B cells, T cells, NK cells, and antigen-presenting cells (APCs). Following activation, mature NK cells release cytokines (IFN-γ, TNF-α, GM-CSF) that induce inflammatory responses; modulate monocytes, dendritic cell, and granulocyte growth and differentiation; as well as influence subsequent adaptive immune responses [14]. IFN-γ secretion by NK cells is important, particularly in early host defense against infection, whereas T lymphocytes become the major source of this cytokine during the adaptive immune response. IFN-γ production by NK cells has been shown to suppress tumor growth by inhibiting angiogenesis [15] and to promote antiviral mechanisms by enhancing nitric oxide production [16]. IFN-γ may also induce resistance to infection by acting on antigen APCs, particularly monocytes/macrophages and dendritic cells, to protect them from infection and to promote their function for stimulation of adaptive immunity [17]. Aberrant IFN-γ expression is associated with a number of autoinflammatory and autoimmune diseases [18].

Using the rat as an animal model, it has been shown that adult rats exposed to ethanol during their fetal life had significant alteration in the physiological rhythms of IFN-γ that was associated with decreased NK cell cytotoxic activity in the spleen [12]. Additionally, it has been shown that replenishment of POMC neurons by neuronal transplants in the hypothalamus prevents stress hyperresponse and increased NK cell cytolytic function and IFN-γ levels in the plasma of adult fetal alcohol-exposed rats [19–21]. However, it is not apparent why the fetal alcohol exposure effect on IFN-γ production persists into the adulthood of fetal alcohol-exposed offspring. Epigenetic changes are now being considered as potential mechanisms of the long-term effects of many toxicants when individuals are exposed to them during development [22]. Hence, by using the well-established animal model, we determined whether fetal alcohol exposure incites epigenetic marks and causes alteration in IFN-γ production in the spleen during the adult period. Furthermore, we tested whether the fetal alcohol exposure-induced epigenetic mark on *Ifn-ɣ* gene propagates through multiple generations, since transgenerational transmission of fetal alcohol effects on some genes have been demonstrated [23–27].

## Methods

### Animals

All rat studies were performed with approved protocols in compliance with the Association for the Assessment and Accreditation of Laboratory Animal Care and Rutgers University. Fisher 344 strain rats were obtained from Harlan Laboratories (Indianapolis, IN) and housed in controlled conditions at a constant temperature of 22 °C, with 12-hour light/dark cycles throughout the study. These rats were bred in our animal facility and used for this study. On gestational days (GD) 7 through 21, rats were fed with rat chow ad libitum (AD), a liquid diet containing ethanol (AF; 1.7–5.0% v/v from GD7–10 and 6.7% v/v from GD11–21; Bioserve Inc., Frenchtown, NJ) or pair-fed (PF; Bioserve) an isocaloric liquid control diet (with alcohol calories replaced by maltose-dextrin). Previous studies have shown that the peak blood ethanol concentration is achieved in the range of 120–150 mg/dl in pregnant dams fed with this ethanol-containing liquid diet [28]. The offspring from these three groups of rats were designated as AD, AF, and PF groups. AF and PF litters were cross-fostered, and the litter size was maintained at 8 pups/dam. Only one pup from each litter was used in an experimental measure. Transgenerational studies were conducted by breeding AF, PF, or AD rats with control animals of the opposite gender to produce two germ lines. We generated a male germ line (AFM or PFM) by breeding male (AF or PF) rats and their male offspring with control (AD) females and a female germ line (AFF or PFF) by breeding female (AF or PF) rats and their female offspring with control (AD) males. All rats were sacrificed at 60–90 days after birth, and splenic tissues were collected for further experimentation.

### Real time PCR for gene expression measurements

Gene expression levels of *Ifn-ɣ* in rat spleen tissue were measured by quantitative RT-PCR (SYBR green assay). Total RNA from the spleen was extracted using an RNeasy kit (Qiagen, Valencia, CA). Total RNA (1 μg) was converted to first-strand complementary DNA (cDNA) using a high-capacity cDNA reverse transcription kit (Life Technologies, Carlsbad, CA, USA). The primer sequences used for the study are given in Table 1. Real-time quantitative PCR was performed at 95 °C for 5 min followed by 40 cycles of 95 °C for 15 s, 60 °C for 30 s, and 72 °C for 40 s using the Applied Biosystems 7500 Real-time PCR system (Foster City, CA). The quantity of target genes (*Ifn-ɣ*) and three reference genes (*Gapdh, 18S, Rpl19*) were measured using the standard curve method. Target gene expression was normalized with the mean of three reference gene expression levels. We have used eight animals in each treatment group to generate the gene expression data.

**Table 1:** Primer sequences

Primer Name	Sequence
*Ifn-ɣ* FP	5’ AAAGACAACCAGGCCATCAGCAAC 3’
*Ifn-ɣ* RP	5’ TCTGTGGGTTGTTCACCTCGAACT 3’
*Ifn-ɣ* BSP FP	5’ TTATAAGAATGGTATAGGTGGGTA 3’
*Ifn-ɣ* BSP RP	5’ -/5Bio/-AACTAATATATCTTCTCTAAATCAACC 3’
*Ifn-ɣ* seq FP	5’ TTATAAGAATGGTATAGGTG 3’
*Gapdh* FP	5’ AGACAGCCGCATCTTCTTGT 3’
*Gapdh* RP	5’ CTTGCCGTGGGTAGAGTCAT 3’
18*S* FP	5’ GTAACCCGTTGAACCCCATT 3’
18*S* RP	5’ CCATCCAATCGGTAGTAGCG 3’
*Rpl*-19 FP	5’ AATCGCCAATGCCAACTCTCG 3’
*Rpl*-19 RP	5’ TGCTCCATGAGAATCCGCTTG 3’

*FP* forward primer, *BSP FP* bisulfite sequencing forward primer, *BSP RP* bisulfite sequencing reverse primer, *5Bio* 5’end biotin labeled, *seq FP* sequencing forward primer

### Western blot analysis for protein measurement

Protein levels of IFN-ϒ were determined by western blot analysis. Total protein from spleen tissue was extracted, and the concentration was measured by protein assay reagent (Bio-Rad Laboratories, Herculus, CA). About 50 μg of total protein was run in 12% SDS PAGE and transferred to PVDF membranes (GE Health Care, Piscataway, NY) at 30 V overnight at 4 °C. The membranes were blocked in 5% non-fat dry milk-TBS-0.1% Tween 20 (TBST) at room temperature for 3 h. The membranes were incubated with primary antibodies in the same blocking buffer at 4 °C overnight. The primary antibodies used were rabbit polyclonal anti-interferon gamma antibody (EPR1108;1:1000; cat#ab133566; Abcam, Cambridge, MA) and mouse anti-β-actin monoclonal antibody (JLA20; cat# CP01; 1:5000, Calbiochem, Billerica, MA). The membranes were washed in TBST and then incubated with corresponding horseradish peroxidase (HRP) conjugated secondary antibody (Vector Laboratories, Burlingame, CA, USA) at room temperature for 1 h. The membranes were washed in TBST and incubated with ECL reagent (Thermo Fisher Scientific, Waltham, MA) and were developed on film by autoradiography. The protein band intensities were determined by Image Studio Lite software (Licor, Lincoln, NE). IFN-ϒ protein band intensity was normalized with corresponding β-actin. We have used six animals in each treatment group to generate the protein expression data.

### Ifn-ɣ promoter DNA methylation by pyrosequencing assay

DNA methylation at the specific CpG site was confirmed by pyrosequencing assay. Briefly, 1 μg of genomic DNA was subjected to bisulfite conversion using an EZ DNA methylation kit (Zymo Research, Orange, CA). Regions of interest were amplified from bisulfite-treated genomic DNA using a PyroMark PCR kit (Qiagen) with forward and biotin-labeled reverse primer as per the instructions from the manufacturer. Biotinylated PCR product was mixed with streptavidin beads and annealed with sequencing primer. Streptavidin-bound biotinylated PCR product was captured using a vacuum filtration sample transfer device (Qiagen). Sequencing was performed using PyroMark Gold Q96 CDT reagents (Qiagen) on a PSQ HS96A model pyrosequencing machine (Qiagen) as per the instructions from the manufacturer. In the pyrosequencing study, we analyzed one control C in a non-CpG background for efficient C to T conversion. The percent methylation was calculated as follows:

% of methylation = % C remaining as C in the target CpGX control C➔T %.

This is for all DNA methylation analysis in each group (*n* = 8) animals were used.

### Statistical analysis

Data were analyzed using Prism 5.0 (GraphPad Software). The data shown in the figures are mean + SEM. The significant differences between different treatment groups were assessed with one-way analysis of variance (ANOVA) with post-hoc analysis using the Newman Keuls post-hoc test. *P* < 0.05 was considered significant. The significant differences between different treatment groups with two variables were assessed with two-way ANOVA with the Bonferroni post-hoc test. *P* < 0.05 was considered significant. F statistics and *P* values of data are presented in Supplement Table 1.

## Results

### Effects of fetal alcohol exposure on *Ifn-ɣ* gene expression, promoter DNA methylation, and protein levels in the spleen of male and female rat offspring

**Fig. 1:**
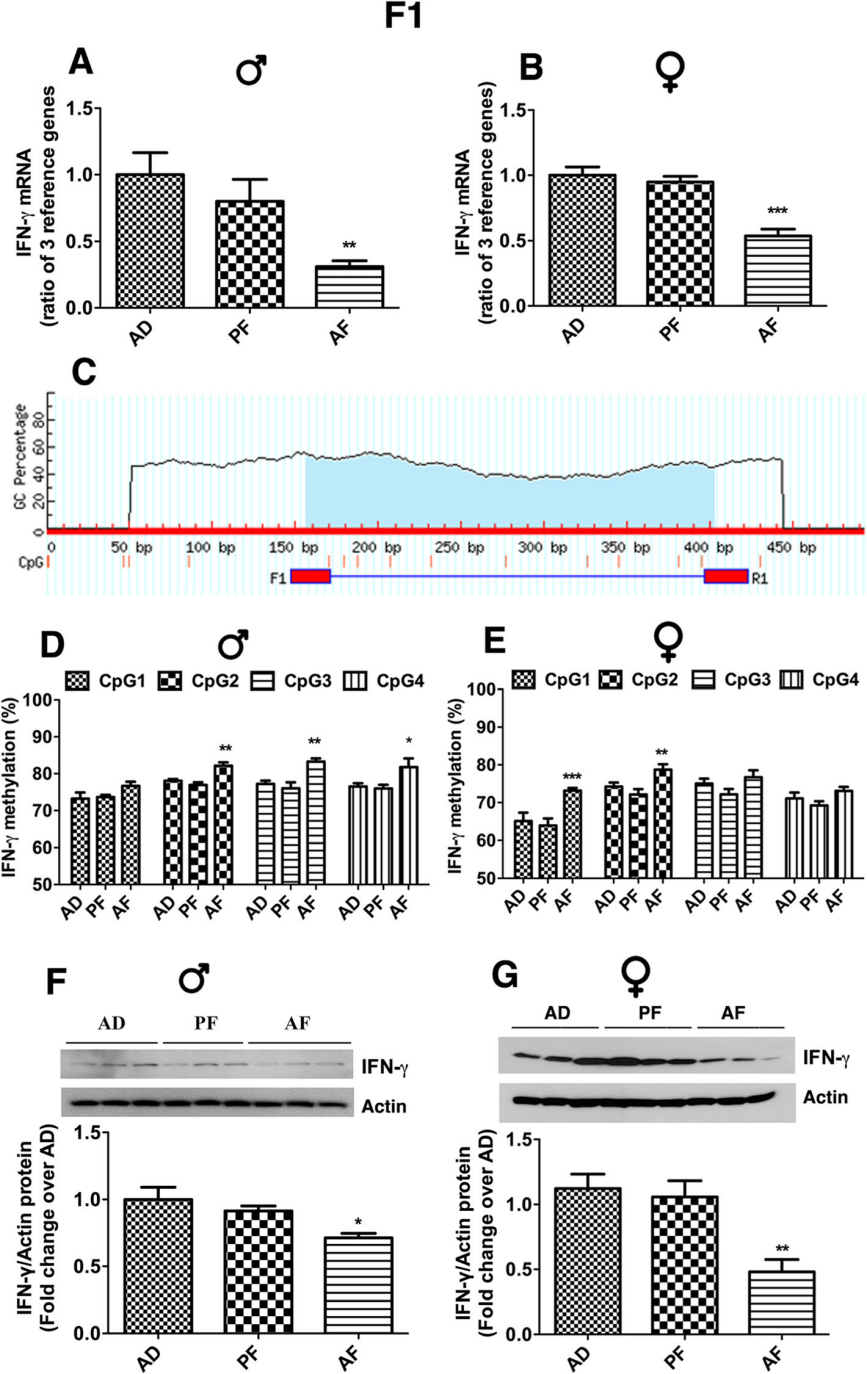
Effects of fetal alcohol exposure on *Ifn-ɣ* gene in the spleen of F1 adult offspring. Changes in the levels of *Ifn-ɣ* mRNA (a, b), *Ifn-ɣ* promoter DNA methylation at various CpGs (**c**–**e**), and IFN-*ɣ* protein (**f**, **g**) in the spleen of male (♂) and female (♀) fetal alcohol-exposed (AF) and control (AD and PF) rat offspring. **a** and **b***Ifn-ɣ* mRNA levels were measured by quantitative RT-PCR. Data are mean + SEM (*n* = 8) and were analyzed using one-way ANOVA with the Newman-Keuls post-hoc test; ** *P* < 0.01, *** *P* < 0.001 between AF and controls. **c** A schematic diagram of rat IFN-ϒ promoter CpG island identified by the Urogene MethPrimer web tool (http://www.urogene.org/methprimer). **d** and **e**. *Ifn-ɣ* promoter CpG methylation changes were measured by pyrosequencing analysis. Four different CpGs of the IFN-ϒ promoter region—CpG1 (-322), CpG2 (-313), CpG3 (-305), and CpG4 (-285) were analyzed. Data are mean + SEM (*n* = 8) and were analyzed using two-way ANOVA with the Bonferroni post-hoc test; * *P* < 0.05, ** *P* < 0.01, and *** *P* < 0.001 between AF and controls of the same CpG. **f** and **g** IFN-ϒ protein levels in spleen samples were measured by western blotting. Representative blots for IFN-ϒ and β-actin are shown in the upper panel and quantification measurements were represented as a histogram in the lower panel. Data are mean + SEM (*n* = 6) and were analyzed using one-way ANOVA with the Newman-Keuls post-hoc test; * *P* < 0.05, ** *P* < 0.01, and *** *P* < 0.001 between AF and controls. F statistics and *P* values of data shown in figures were presented in Supplementary Table 1

We used isogeneic Fischer 344 rats and a well-established liquid diet model of alcohol feeding in pregnant rats between days 7 through 21 of pregnancy, which is equivalent to the part of the first and whole second trimesters of human pregnancy. This model of alcohol feeding in rats is known to raise blood levels of alcohol in the range of 120–150 mg/dl [28] and produce offspring with endophenotypes similar to those found in human fetal alcohol spectrum disorders, such as anxiety behaviors, stress hyperresponsiveness, and metabolic diseases [29–32]. Determination of *Ifn-ɣ* mRNA levels expressed by the ratio of various housekeeping genes in the spleen revealed that they were similar in AD and PF male and female rats during adulthood, suggesting a minimum impact of the liquid diet feeding paradigm on *Ifn-ɣ* expression (Fig. 1a, b). However, *Ifn-ɣ* mRNA levels in the spleen were lower in AF compared to AD and PF rats, without any sex difference (Fig. 1a, b). These data indicate that fetal alcohol exposure results in reduced expression of *Ifn-ɣ* in the spleen of both male and female offspring.

Global or site-specific methylation of CpG sites near and within regulatory regions of genes is often associated with transcriptional inactivity and gene suppression [33]. We used the Urogene MethPrimer web tool (http://www.urogene.org/methprimer) to analyze *Ifn-ɣ* promoter and found the CpG island extended upstream of the transcriptional start site (Fig. 1c). The methylation status of four CpG dinucleotides (CpG1, -322; CpG2, -313; CpG3, -305; CpG4, -285) in the proximal promoter of the *Ifn-ɣ* gene was determined by the pyrosequencing technique. We calculated % of methylation as (% C remaining as C in the target CpG X control C➔T %) and found that the percent of methylation is elevated by fetal alcohol feeding in all CpGs sites of *Ifn-ɣ* gene in both sexes, but CpG2, CpG3, and CpG4 are significantly higher in male offspring and CpG1 and CpG2 are significantly higher in female offspring, when compared with those in AD and PF rats (Fig. 1d, e). Overall, these data indicate that fetal alcohol alters methylation of *Ifn-ɣ* promoter DNA in adulthood, even though the exposure to ethanol ended at the prenatal period.

Like the effects on *Ifn-ɣ* mRNA levels, fetal alcohol exposure reduced IFN-ϒ protein levels in the spleen in both male and female rats (Fig. 1f, g). Alcohol treatment did not affect the level of housekeeping protein actin in spleen samples, suggesting very little impact of alcohol feeding on general protein synthesis. These data suggest that fetal alcohol exposure produces a long-lasting effect on the spleen such that the adult expression of the IFN-ϒ protein is reduced.

### Fetal alcohol induced transgenerational changes in *Ifn-ɣ* gene expression, promoter DNA methylation, and protein levels in the spleen of male and female offspring

**Fig. 2:**
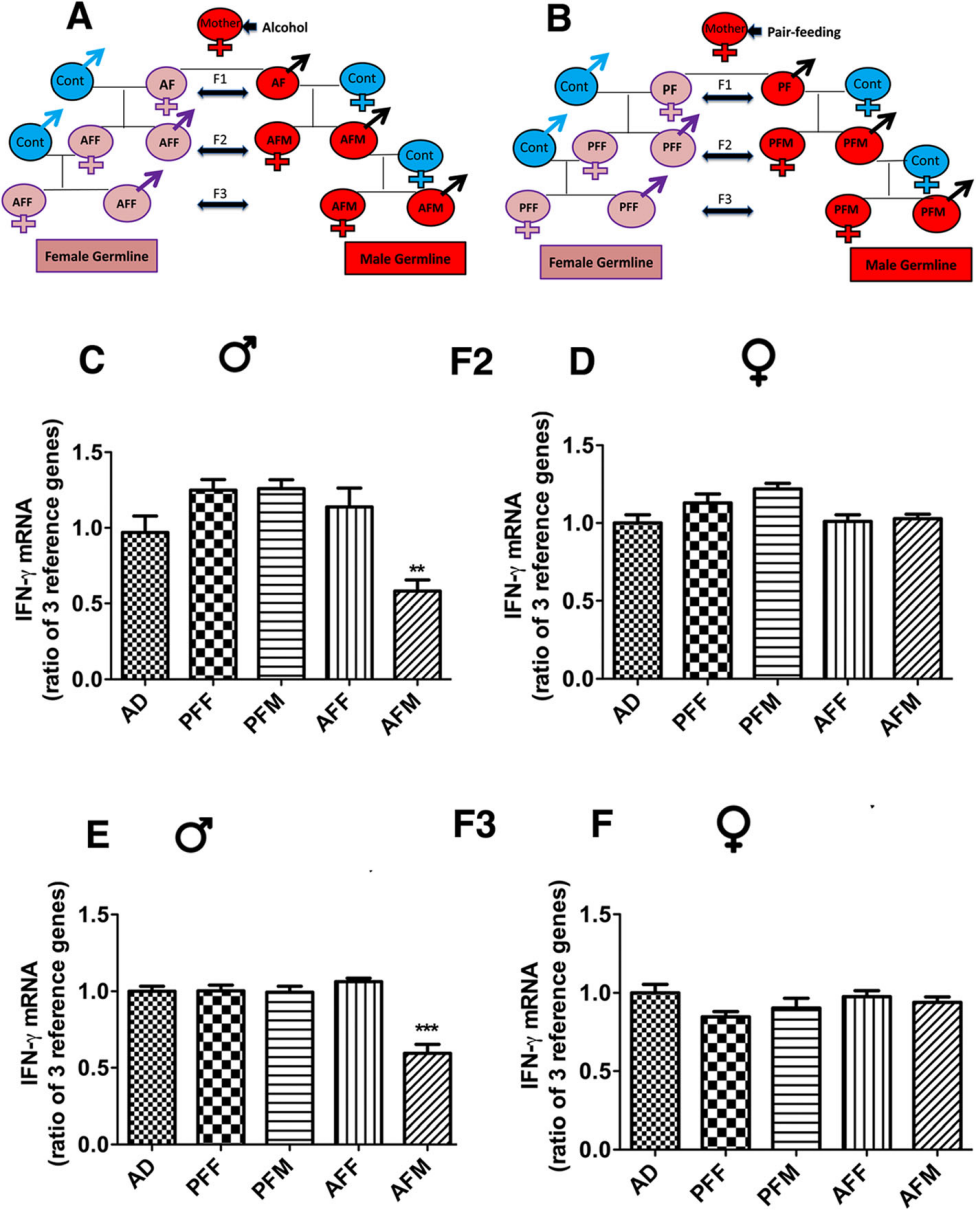
Transgenerational changes in *Ifn-ɣ* mRNA expression in the spleen after alcohol feeding in pregnant female rats. A schematic diagram to indicate how F2 and F3 male germ line (AFM) and female germ line (AFF) of fetal alcohol-exposed (**a**) and male germ line (PFM) and female germ line (PFF) of pair-fed control offspring (**b**) were generated. *Ifn-* mRNA levels in the spleen tissues of F2 (**c**, **d**) and F3 (**e**, **f**) male and female rat offspring from different germ lines were measured by quantitative RT-PCR. Data are mean + SEM (*n* = 8) and were analyzed using one-way ANOVA with Newman-Keuls post-hoc test; ** *P* < 0.01, *** P < 0.001 between AFM and controls (AD, PFM). F statistics and *P* values of data shown in figures were presented in Supplementary Table 1

Whether the fetal alcohol effect is transmitted transgenerationally was tested by studying *Ifn-ɣ* gene and protein expression and DNA methylation in the spleen of F2 and F3 male (AFM) and female germ lines (AFF). We produced two different germ lines—a male germ line by breeding male fetal alcohol-exposed rats and their male offspring with normal females, and a female germ line by breeding female fetal alcohol-exposed rats and their female offspring with normal males as we have previously described (Fig. 2a). We also produced male (PFM) and female germ lines (PFF) of control-fed rats (Fig. 2b). We measured the expression of *Ifn-ɣ* in F2 and F3 male and female germ lines to determine whether the fetal alcohol effect is transmitted through successive generations. Like in the F1 generation, the F2 male progeny of male germ lines (AFM) showed a significant reduction in *Ifn-ɣ* mRNA levels in the spleen as compared to the corresponding control groups (Fig. 2c). F2 female progeny of male germ lines (AFM) did not show any significant effect on *Ifn-ɣ* mRNA levels in the spleen (Fig. 2c). Also, F2 male and female progeny of the female germ line (AFF) did not show any changes in *Ifn-ɣ* mRNA levels, as compared to controls (Fig. 2d). In the F3 generation, like in the F2 progeny, the F3 male progeny of the male germ line (AFM) showed a reduction in *Ifn-ɣ* mRNA levels (Fig. 2e). Other treatment groups did not differ from the control groups (Fig. 2e, f).

**Fig. 3:**
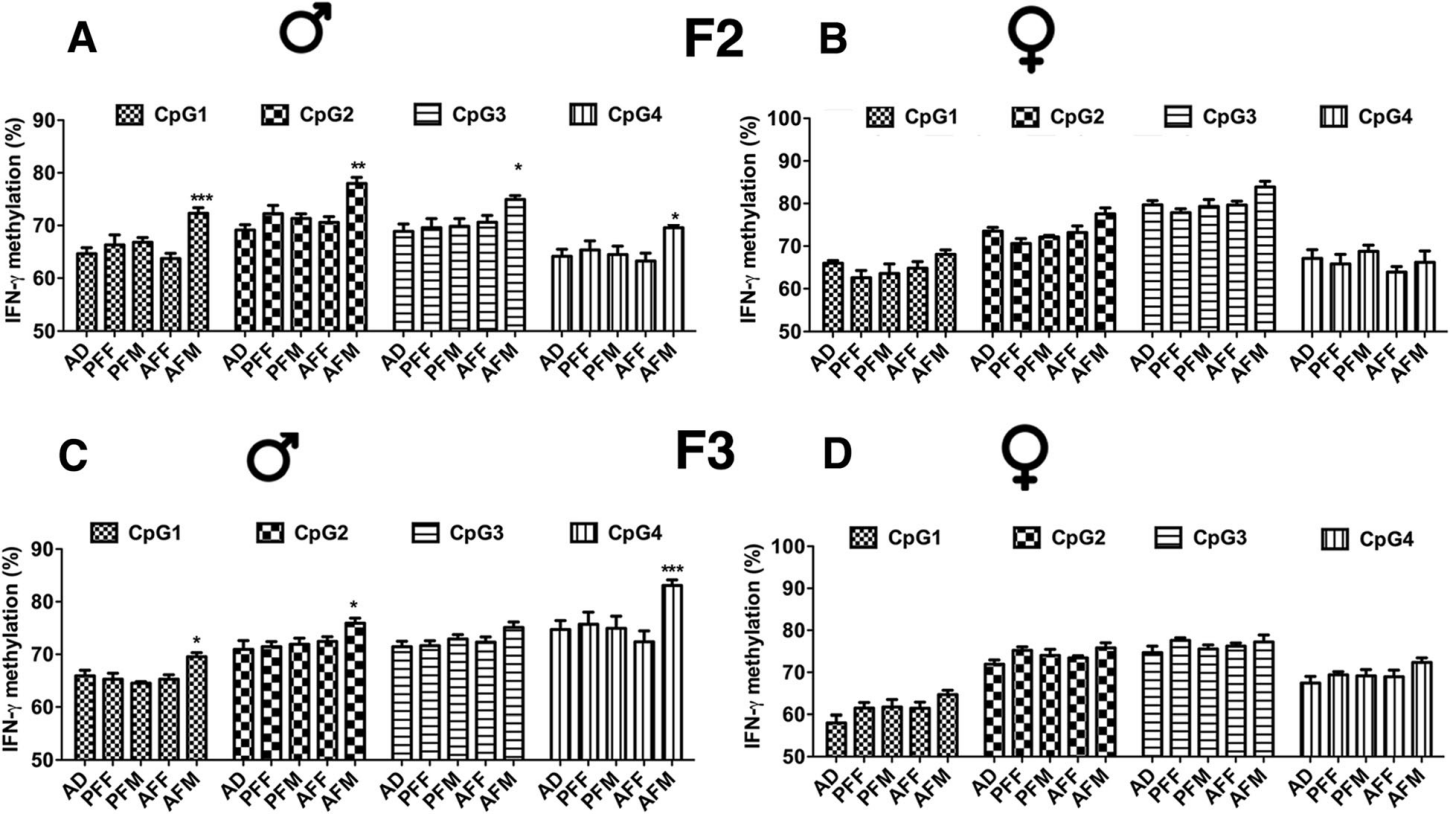
Transgenerational changes in *Ifn-e* methylation in the spleen after alcohol feeding in pregnant female rats. **I**FN-pleen after alcohol feeding inwere determined by pyrosequencing in the spleen of F2 male (**a**), F2 female (**b**), F3 male (**c**), and F3 female (**d**) rat offspring from different germ lines as defined in Fig. 2a. Four different CpGs of the IFN- different CpGs o—CpG1 (-322), CpG2 (-313), CpG3 (-305), and CpG4 (-285) were analyzed. Data are mean + SEM (*n* = 7–8) and were analyzed using two-way ANOVA with the Bonferroni post-hoc test. * *P* < 0.05, ** *P* < 0.01, *** *P* < 0.001 between AFM vs. controls (AD, PFM). F statistics and P values of data shown in figures were presented in Supplementary Table 1

In order to determine whether fetal alcohol-induced epigenetic modification of *Ifn-ɣ* promoter is transmitted through successive generations, we evaluated *Ifn-ɣ* promoter DNA methylation changes in the spleen of alcohol-fed and pair-fed F2 and F3 progeny of male and female germ lines (animal breeding is as shown in Fig. 2a, b). We analyzed four different CpGs in the *Ifn-ɣ* promoter CpG island using pyrosequencing (as shown in Fig. 1c). In the F2 generation, *Ifn-ɣ* promoter DNA methylation levels in the spleen were significantly increased in all four CpGs (CpG1–4) in male offspring of the male germ line (AFM) and not in the female germ line (AFF) compared to controls (Fig. 3a, b). *Ifn-ɣ* promoter DNA methylation levels in the spleen did not change in the female offspring of the male or female germ lines (Fig. 3b). The F3 male progeny of the male germ line (AFM) also showed a significant increase in *Ifn-ɣ* promoter DNA methylation in three CpGs (CpG1, CpG2, and CpG4) compared to corresponding controls (Fig. 3c). However, the F3 female progeny of the male germ line and the F3 male and female progeny of the female germ line did not show any change in *Ifn-ɣ* promoter DNA methylation (Fig. 3c, d).

**Fig. 4:**
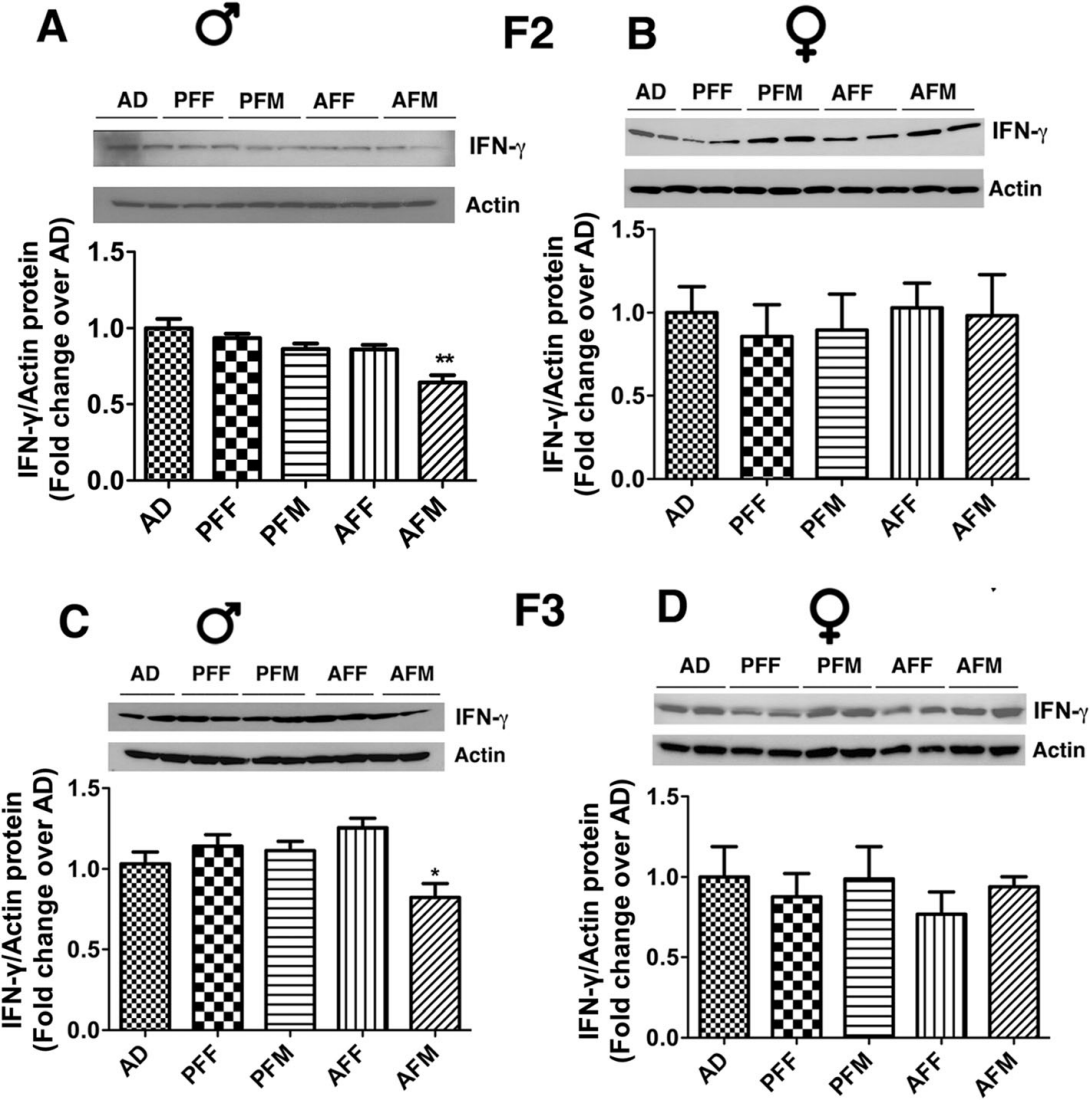
Transgenerational changes in *IFN- en*protein levels in the spleen after alcohol feeding in pregnant female rats**. I**FN-ϒ protein levels in the spleens of F2 and F3 male (**a**, **c**) and female (**b**, **d**) rat offspring from different germ lines as defined in Fig. 2a. IFN-ɣ protein levels in the spleens were measured by western blotting. Representative blots for IFN-ϒ, β-actin were shown in the upper panel and quantification data normalized with β-actin were represented as a histogram in the lower panel for each figure. Data are mean + SEM (*n* = 6–7) and were analyzed using one-way ANOVA with the Newman-Keuls post-hoc test. * *P* < 0.05, ** *P* < 0.01 between AFM vs. AD or PFM. F statistics and P values of data shown in figures were presented in Supplementary Table 1

IFN-ϒ protein levels were also determined in spleen samples of F2 and F3 progeny of male and female germ lines. IFN-ϒ protein levels in spleen samples of F2 and F3 offspring mirrored the transgenerational effects of fetal alcohol observed in *Ifn-ɣ* mRNA levels in spleen samples and verified the significant inhibitory effect of fetal alcohol on the production of this cytokine in the F2 and F3 male progeny of the male germ line (Fig. 4a, c). The female AF progeny of the male or female germ lines did not show any changes in F2 or F3 generations as compared to those of PF and AD progeny (Fig. 4b, d).

## Discussion

In this study, we showed that fetal alcohol exposure reduced basal expressions of *Ifn-ɣ* mRNA and protein in the spleen during the adult period in both male and female offspring. We also showed here that fetal alcohol exposure induced hypermethylation of the *Ifn-ɣ* gene that persisted in the adult period. This conclusion is based on the findings that the percentage of the cytosine methylation of CpG-rich sites adjacent to the *Ifn-ɣ* gene transcription start site was higher in alcohol-fed animals than in controls. Two (in female) to three CpGs (in male) out of four analyzed showed increased methylation in fetal alcohol exposed offspring, suggesting some specificity of alcohol action towards certain CpG methylation in the promoter region of the *Ifn-ɣ* gene. Although chronic alcohol consumption induces hypomethylation in general, its effect on DNA methylation is gene- and tissue-specific in the developmental stage of exposure [34, 35]. We previously reported that fetal alcohol exposure results in hypermethylation of *Pomc* promoter in the hypothalamus and *D2r* promoter in the pituitary during the adult period [26, 36]. The present data provide associative evidence for the involvement of gene hypermethylation in the suppression of *Ifn-ɣ* mRNA expression in fetal alcohol-exposed rats. Previous studies have shown a similar inverse association between *Ifn-ɣ* gene methylation and *Ifn-ɣ* mRNA expression in oral cancer as well as in vitamin D treatment during fetal growth [37, 38]. Furthermore, it has been shown that demethylation of some of the CpG sites in the promoter of the *Ifn-ɣ* gene increases mRNA expression in CD44(high)CD8+ T cells [39]. Also, site-specific differences in CpG methylation and *Ifn-ɣ* gene expression have been reported in various immune cells [40]. Hence, increased DNA methylation in the *Ifn-ɣ* gene promoter may have been a cause for lowering the *Ifn-ɣ* gene expression.

We also provide the first evidence of perpetuation of fetal alcohol-induced changes in DNA methylation and expression of the *Ifn-ɣ* gene across multiple generations through the male germ line. Studies including prenatal immune-activation-linked traits have been demonstrated in non-exposed offspring (reviewed in [41]). However, most of these traits are transmitted only to direct offspring of exposed individuals. In our animal model, we determined a fetal alcohol-induced immune trait for multiple generations by continuing breeding fetal alcohol-exposed animals and their offspring with non-alcohol treated controls up to the F3 generation. We made distinct male and female germ lines by breeding fetal alcohol-exposed males and their male offspring with normal females, and fetal alcohol-exposed females and their female offspring with normal males. We only found fetal alcohol-induced traits on IFN-ϒ production persisted in the F2 and F3 generations derived from the male germ line. We previously reported fetal alcohol-induced *Pomc* promoter hypermethylation and its endophenotypes are transmitted to the F3 generation only through the male germ line but not through the female germ line [26]. We also provided evidence that sperm DNA methylation changes may be involved in the transgenerational transmission of fetal alcohol effects on the *Pomc* gene [26]. In our present study, FAE-induced *Ifn-ɣ* expression and IFN*-*ɣ protein level changes are transmitted from the F1 generation to the F3 generation only through the male germ line in the male offspring, but no changes were observed in female offspring. FAE-induced *Ifn-ɣ* promoter CpG methylation changes also passed from the F1 to the F3 generation through the male germ line in male progeny. However, in female offspring, this trait was only transmitted to the F2 generation in the male germ line in male progeny. In recent years, there has been growing evidence suggesting that paternal exposure to a low-fat diet induces transgenerational inheritance of metabolic gene expression [42], while traumatic stress induces behavioral and metabolic phenotypes [43]. A few other studies also showed paternal exposure to endocrine-disrupting compounds which caused decreased spermatogenic activity and increased incidence of male infertility, and these effects correlate with DNA methylation patterns in the germ cells and were transmitted through the male germ line [44]. It has been shown that prenatal immune challenge during early/mid-gestation caused some behavioral abnormalities in the offspring, and these traits were transmitted to subsequent generations [45]. Also, prenatal immune-challenge-induced behavioral abnormalities are shown to be transmitted transgenerationally via the paternal lineage [46]. The mechanism for the transgenerational transmission of a physiological trait via the male germ line is currently not well established. Primordial germ cells (PGS), the precursors of eggs and sperm, undergo epigenetic reprogramming involving erasure of cytosine methylation. In mouse, during embryonic days 8–13.5 (E8–13.5), global epigenome resetting takes place as PGS migrate from the yolk sac to the genital ridge [47]. In mammals, epigenetic reprogramming is a major barrier to epigenetic transgenerational inheritance. However, DNA methylation at specific loci resist erasure in PGCs with a potential for epigenetic inheritance. Some of these genetic loci were partially programmed in germ lines and are sensitive to environmental stimuli, and these variations in methylation levels may contribute to transgenerational epigenetic inheritance [48]. A recent study has provided evidence for the implication of sperm microRNAs in the transgenerational inheritance of trauma-induced phenotypes across generations in mice [49]. Whether a similar mechanism is involved in the transgenerational inheritance of fetal alcohol-induced changes in the *Ifn-ɣ* gene needs further investigation.

In summary, our data highlights the novel finding of fetal alcohol-exposure-induced epigenetic changes on *Ifn-ɣ* expression as well as its transmission through successive generations via the male germ line. The exact mechanism of this male-specific transgenerational inheritance of epigenetic modification of *Ifn-ɣ* promoter DNA methylation is currently under investigation. The data of this study may have clinical significance as IFN-γ has become an important therapeutic agent to cure several infections due to its immune-modulatory effect.

## Supplementary information

**Additional file 1: Table S1**. Statistical information (F statistics and p values) of data in Figs. 1, 2, 3, 4.

## Abbreviations

AD: Ad libitum
AFF: Alcohol-fed female germ line
AFM: Alcohol-fed male germ line
AF: Alcohol fed
APCs: Antigen-presenting cells
Gapdh: Glyceraldehyde-3-phosphate dehydrogenase
GD: Gestational day
GM-CSF: Granulocyte-macrophage colony-stimulating factor
HRP: Horseradish peroxidase
IFN-Y: Interferon-ϒ
NK: Natural killer
ANOVA: One-way analysis of variance
PFF: Pair-fed female germ line
PFM: Pair-fed male germ line
PF: Pair fed
POMC: Proopiomelanocortin
RT-PCR: Real-time PCR
Rpl19: Ribosomal protein L19
TBS: Tris-buffered saline
TBST: TBS-0.1% Tween 20
TNF-α: Tumor necrosis factor-α
