# Supplementary material for: Transgenerational inheritance of fetal alcohol exposure adverse effects on immune gene interferon-ϒ
Source: Clin Epigenetics. 2020 May 24;12:70. doi: 10.1186/s13148-020-00859-9 (PMC7245772; doi:10.1186/s13148-020-00859-9)
Supplement: Supplementary file 1 — Additional file 1: Table S1. Statistical information (F statistics and p values) of data in Figs. 1, 2, 3, 4. [file 13148_2020_859_MOESM1_ESM.docx]

**Suppl. Table 1 : Statistical information (F statistics and p values) of data in Figs. 1, 2, 3, 4.**

| Figure | Experiment | F-statistic | p-value |
| --- | --- | --- | --- |
| 1A | One-way ANOVA  Ifn-ϒ mRNA levels in the spleen of F1 male rats (AD, PF, AF)  AD vs AF  PF vs AF  AD vs PF | Alcohol : F (2,21)=6.65 | P<0.01  P<0.01 **  P<0.05 *  p>0.05 (ns) |
| 1B | One-way ANOVA  Ifn-ϒ mRNA levels in the spleen of F1 female rats (AD, PF, AF)  AD vs AF  PF vs AF  AD vs PF | Alcohol : F(2,21)=23.02 | P<0.0001  P<0.001 ***  P<0.001 ***  p>0.05 (ns) |
| 1D | Two-way ANOVA  Ifn-ϒ promoter DNA methylation at various CpGs in F1 male rats (AD, PF,AF)  CpG1 : AD vs AF  PF vs AF  AD vs PF  CpG2 : AD vs AF  PF vs AF  AD vs PF  CpG3 : AD vs AF  PF vs AF  AD vs PF  CpG4 : AD vs AF  PF vs AF  AD vs PF | Alcohol : F(2,78)=23.74  CpGs : F(3,78)=9.53  Interaction: F(6,78)=0.56 | P<0.0001  P<0.0001  P=0.760  p>0.05  p>0.05 ns  p>0.05  p>0.05  p<0.01 **  p>0.05  p<0.01 **  p<0.001 ***  p>0.05  p<0.05 *  p<0.01 **  p>0.05 |
| 1E | Two-way ANOVA  Ifn-ϒ promoter DNA methylation at various CpGs in F1 female rats (AD, PF,AF)  CpG1 : AD vs AF  PF vs AF  AD vs PF  CpG2 : AD vs AF  PF vs AF  AD vs PF  CpG3 : AD vs AF  PF vs AF  AD vs PF  CpG4 : AD vs AF  PF vs AF  AD vs PF | Alcohol : F(2,84)=17.51  CpGs : F(3,84)=17.53  Interaction: F(6,84)=1.15 | P<0.0001  P<0.0001  P=0.342  p<0.001 ***  p<0.001 ***  p>0.05  p>0.05  p<0.01 **  p>0.05  p>0.05  p>0.05 ns  p>0.05  p>0.05  p>0.05 ns  p>0.05 |
| 1F | One-way ANOVA  Ifn-ϒ protein levels in the spleen of F1 male rats (AD, PF, AF)  AD vs AF  PF vs AF  AD vs PF | Alcohol : F(2,24)=6.35 | P<0.01  P<0.01 **  P<0.05 *  p>0.05 (ns) |
| 1G | One-way ANOVA  Ifn-ϒ protein levels in the spleen of F1 female rats (AD, PF, AF)  AD vs AF  PF vs AF  AD vs PF | Alcohol : F(2,27)=10.23 | P<0.001  P<0.01 **  P<0.001 ***  p>0.05 (ns) |
| 2C | One-way ANOVA  Ifn-ϒ mRNA levels in the spleen of F2 male rats (AD, PFF, PFM, AFF, AFM)  AD vs AFM  PFM vs AFM  AD vs AFF  PFF vs AFF  AD vs PFF  AD vs PFM  PFF vs PFM | Alcohol : F(4,36)=9.24 | P<0.0001  P<0.01 **  P<0.01 **  p>0.05 ns  p>0.05 ns  p>0.05  p>0.05 ns  p>0.05 |
| 2D | One-way ANOVA  Ifn-ϒ mRNA levels in the spleen of F2 female rats (AD, PFF, PFM, AFF, AFM)  AD vs AFM  PFM vs AFM  AD vs AFF  PFF vs AFF  AD vs PFF  AD vs PFM  PFF vs PFM | Alcohol : F(4,35)=4.43 | P<0.01  p>0.05 ns  p<0.05 *  p>0.05 ns  p>0.05 ns  p>0.05  p<0.05 ns  p>0.05 |
| 2E | One-way ANOVA  Ifn-ϒ mRNA levels in the spleen of F3 male rats (AD, PFF, PFM, AFF, AFM)  AD vs AFM  PFM vs AFM  AD vs AFF  PFF vs AFF  AD vs PFF  AD vs PFM  PFF vs PFM | Alcohol : F(4,35)=22.73 | P<0.0001  P<0.001 ***  P<0.001 ***  p>0.05 ns  p>0.05 ns  p>0.05  p>0.05 ns  p>0.05 |
| 2F | One-way ANOVA  Ifn-ϒ mRNA levels in the spleen of F3 female rats (AD, PFF, PFM, AFF, AFM)  AD vs AFM  PFM vs AFM  AD vs AFF  PFF vs AFF  AD vs PFF  AD vs PFM  PFF vs PFM | Alcohol : F(4,35)=1.71 | p>0.05  p>0.05 ns  p>0.05 ns  p>0.05 ns  p>0.05 ns  p>0.05  p>0.05 ns  p>0.05 |
| 3A | Two-way ANOVA  Ifn-ϒ promoter DNA methylation at various CpGs in F2 male rats (AD, PFF, PFM, AFF, AFM)  CpG1 : AD vs AFM  PFM vs AFM  AD/PFF vs AFF  AD vs PFF/PFM  CpG2 : AD vs AFM  PFM vs AFM  AD/PFF vs AFF  AD vs PFF/PFM  CpG3 : AD vs AFM  PFM vs AFM  AD/PFF vs AFF  AD vs PFF/PFM  CpG4 : AD vs AFM  PFM vs AFM  AD/PFF vs AFF  AD vs PFF/PFM | Alcohol : F(4,28)=17.7  CpGs : F(3,128)=30.64  Interaction:F(12,128)=0.465 | P<0.0001  P<0.0001  P=0.932  P<0.001 ***  p>0.001 ***  p>0.05 (ns)  p>0.05 (ns)  p<0.001 ***  p<0.01 **  p>0.05 (ns)  p>0.05 (ns)  p<0.01 **  p<0.05 *  p>0.05 (ns)  p>0.05 (ns)  p<0.05 *  p<0.05 *  p>0.05 (ns)  p>0.05 (ns) |
| 3B | Two-way ANOVA  Ifn-ϒ promoter DNA methylation at various CpGs in F2 female rats (AD, PFF, PFM, AFF, AFM)  CpG1 : AD vs AFM  PFM vs AFM  AD/PFF vs AFF  AD vs PFF/PFM  CpG2 : AD vs AFM  PFM vs AFM  AD/PFF vs AFF  AD vs PFF/PFM  CpG3 : AD vs AFM  PFM vs AFM  AD/PFF vs AFF  AD vs PFF/PFM  CpG4 : AD vs AFM  PFM vs AFM  AD/PFF vs AFF  AD vs PFF/PFM | Alcohol : F(4,136)=5.41  CpGs : F(3,136)=104  Interaction : F(12,136)=1.07 | P<0.001  P<0.0001  P=0.395  P>0.05 ns  P>0.05 ns  P>0.05 (ns)  p>0.05 (ns)  P>0.05 ns  P>0.05 ns  P>0.05 (ns)  p>0.05 (ns)  P>0.05 ns  P>0.05 ns  P>0.05 (ns)  p>0.05 (ns)  P>0.05 ns  P>0.05 ns  P>0.05 (ns)  p>0.05 (ns) |
| 3C | Two-way ANOVA  Ifn-ϒ promoter DNA methylation at various CpGs in F3 male rats (AD, PFF, PFM, AFF, AFM)  CpG1 : AD vs AFM  PFM vs AFM  AD/PFF vs AFF  AD vs PFF/PFM  CpG2 : AD vs AFM  PFM vs AFM  AD/PFF vs AFF  AD vs PFF/PFM  CpG3 : AD vs AFM  PFM vs AFM  AD/PFF vs AFF  AD vs PFF/PFM  CpG4 : AD vs AFM  PFM vs AFM  AD/PFF vs AFF  AD vs PFF/PFM | Alcohol : F(4,126)=13.01  CpGs : F(3,126)=54.01  Interaction : F(12,126)=1.19 | P<0.0001  P<0.0001  P=0.298  p>0.05 ns  p<0.05 *  p>0.05 (ns)  p>0.05 (ns)  p<0.05 *  p>0.05 ns  p>0.05 (ns)  p>0.05 (ns)  p>0.05 ns  p>0.05 ns  p>0.05 (ns)  p>0.05 (ns)  p<0.001 ***  p<0.001 ***  p>0.05 (ns)  p>0.05 (ns) |
| 3D | Two-way ANOVA  Ifn-ϒ promoter DNA methylation at various CpGs in F3 female rats (AD, PFF, PFM, AFF, AFM)  CpG1 : AD vs AFM  PFM vs AFM  AD/PFF vs AFF  AD vs PFF/PFM  CpG2 : AD vs AFM  PFM vs AFM  AD/PFF vs AFF  AD vs PFF/PFM  CpG3 : AD vs AFM  PFM vs AFM  AD/PFF vs AFF  AD vs PFF/PFM  CpG4 : AD vs AFM  PFM vs AFM  AD/PFF vs AFF  AD vs PFF/PFM | Alcohol : F(4,133)=5.99  CpGs : F(3,133) =126.3  Interaction : F(12,133)=0.35 | P<0.001  P<0.0001  P=0.977  P<0.01 **  p>0.05 ns  p>0.05 (ns)  p>0.05 (ns)  p>0.05 (ns)  p>0.05 (ns)  p>0.05 (ns)  p>0.05 (ns)  p>0.05 (ns)  p>0.05 (ns)  p>0.05 (ns)  p>0.05 (ns)  p<0.05 *  p>0.05 (ns)  p>0.05 (ns)  p>0.05 (ns) |
| 4A | One-way ANOVA  Ifn-ϒ protein levels in the spleen of F2 male rats (AD, PFF, PFM, AFF, AFM)  AD vs AFM  PFM vs AFM  AD vs AFF  PFF vs AFF  AD vs PFF  AD vs PFM  PFF vs PFM | Alcohol : F(4,25)=9.95 | P<0.0001  P<0.001 ***  P<0.01 **  p>0.05 (ns)  p>0.05 (ns)  p>0.05  p>0.05 (ns)  p>0.05 |
| 4B | One-way ANOVA  Ifn-ϒ protein levels in the spleen of F2 female rats (AD, PFF, PFM, AFF, AFM)  AD vs AFM  PFM vs AFM  AD vs AFF  PFF vs AFF  AD vs PFF  AD vs PFM  PFF vs PFM | Alcohol : F(4,30)=0.196 | p>0.05  p>0.05 (ns)  p>0.05 (ns)  p>0.05 (ns)  p>0.05 (ns)  p>0.05  p>0.05 (ns)  p>0.05 |
| 4C | One-way ANOVA  Ifn-ϒ protein levels in the spleen of F3 male rats (AD, PFF, PFM, AFF, AFM)  AD vs AFM  PFM vs AFM  AD vs AFF  PFF vs AFF  AD vs PFF  AD vs PFM  PFF vs PFM | Alcohol : (4,30)=5.12 | P<0.01  P<0.05 *  P<0.05 *  p>0.05 (ns)  p>0.05 (ns)  p>0.05  p>0.05 (ns)  p>0.05 |
| 4D | One-way ANOVA  Ifn-ϒ protein levels in the spleen of F3 female rats (AD, PFF, PFM, AFF, AFM)  AD vs AFM  PFM vs AFM  AD vs AFF  PFF vs AFF  AD vs PFF  AD vs PFM  PFF vs PFM | Alcohol : (4,30)=0.328 | p>0.05  p>0.05 (ns)  p>0.05 (ns)  p>0.05 (ns)  p>0.05 (ns)  p>0.05  p>0.05 (ns)  p>0.05 |

(*, **, *** significant at p<0.05, 0.01, 0.001. ns=no significance)
